# Distinct Types of Disorder in the Human Proteome: Functional Implications for Alternative Splicing

**DOI:** 10.1371/journal.pcbi.1003030

**Published:** 2013-04-25

**Authors:** Recep Colak, TaeHyung Kim, Magali Michaut, Mark Sun, Manuel Irimia, Jeremy Bellay, Chad L. Myers, Benjamin J. Blencowe, Philip M. Kim

**Affiliations:** 1 The Donnelly Centre, University of Toronto, Toronto, Ontario, Canada; 2 Banting and Best Department of Medical Research, University of Toronto, Toronto, Ontario, Canada; 3 Department of Computer Science, University of Toronto, Toronto, Ontario, Canada; 4 Department of Computer Science and Engineering, University of Minnesota, Minneapolis, Minnesota, United States of America; 5 Department of Molecular Genetics, University of Toronto, Toronto, Ontario, Canada; Center for Genomic Regulation, Spain

## Abstract

Intrinsically disordered regions have been associated with various cellular processes and are implicated in several human diseases, but their exact roles remain unclear. We previously defined two classes of conserved disordered regions in budding yeast, referred to as “flexible” and “constrained” conserved disorder. In flexible disorder, the property of disorder has been positionally conserved during evolution, whereas in constrained disorder, both the amino acid sequence and the property of disorder have been conserved. Here, we show that flexible and constrained disorder are widespread in the human proteome, and are particularly common in proteins with regulatory functions. Both classes of disordered sequences are highly enriched in regions of proteins that undergo tissue-specific (TS) alternative splicing (AS), but not in regions of proteins that undergo general (i.e., not tissue-regulated) AS. Flexible disorder is more highly enriched in TS alternative exons, whereas constrained disorder is more highly enriched in exons that flank TS alternative exons. These latter regions are also significantly more enriched in potential phosphosites and other short linear motifs associated with cell signaling. We further show that cancer driver mutations are significantly enriched in regions of proteins associated with TS and general AS. Collectively, our results point to distinct roles for TS alternative exons and flanking exons in the dynamic regulation of protein interaction networks in response to signaling activity, and they further suggest that alternatively spliced regions of proteins are often functionally altered by mutations responsible for cancer.

## Author Summary

A protein's cellular and molecular function is typically determined by its folded structure. However, a large fraction of proteomes lack stably folded structure. These regions are referred to as intrinsically disordered. Protein disorder has largely been understudied, although it is emerging to have numerous important functions in a cell. Similarly, although alternative splicing (AS) is well established as an important regulatory layer of metazoan gene expression, its specific roles at the protein level are not well understood. Others and we recently have provided evidence that tissue-regulated AS likely plays a widespread role in the control of protein-protein interactions. In the present study, we investigate how two different classes of conserved protein disorder may contribute distinct functions in relation to roles of regulated alternative exons in the dynamic remodeling of interaction networks. We also investigate the distribution of cancer causing mutations in regulated and other alternatively spliced regions of proteins.

## Introduction

While it is well established that a protein's three-dimensional structure determines its function, a large fraction of proteins and protein regions lack stable structure. Such intrinsically disordered proteins contain extended regions that do not fold into a native fixed conformation [1]. These disordered regions are widespread across the tree of life, particularly in eukaryotes [2]. For example, amino acids comprising approximately 30–40% of the human proteome are predicted to reside within disordered regions [3]. Many different functions have been ascribed to disordered proteins. For instance, they have been shown to carry out regulatory functions associated with signal transduction and molecular recognition, including transcription, protein phosphorylation, mRNA metabolism, RNA processing, translation, chaperone activity and regulation of the cell cycle [1], [4], [5].

Alternative splicing (AS) and post-translational modification such as phosphorylation are known to regulate and diversify the functions of proteins and are thought to partly account for the increased complexity of metazoan species. Human alternatively spliced exons are enriched in regions of intrinsic disorder, presumably to provide functional and regulatory diversity while avoiding disruption to core protein structure [3], [6], [7]. Moreover, we and others have recently shown that tissue-regulated alternative exons are enriched in highly disordered regions of proteins where they frequently modulate interactions in protein-protein interaction networks [8]–[10]. In addition, disordered regions often harbor linear motifs that mediate recognition functions and therefore can be considered as a class of functional domain [11], [12].

Finally, intrinsic disorder is abundant among proteins associated with various human diseases such as cancer, cardiovascular disease, amyloidoses, diabetes, neurodegenerative diseases and others [13]. Furthermore, highly connected proteins in “diseasome” networks are enriched in disorder [14]. However, due to the wide range of roles of disordered proteins it has been difficult to ascribe specific functions to disordered regions.

In order to better understand the roles of intrinsic disorder, we previously developed a method to analyze the conservation of intrinsic disorder across the yeast clade [15]. Over large regions of proteins, the property of disorder is highly conserved, i.e., the same residues are disordered in most orthologous proteins. Additionally, the underlying amino acid sequence of the disordered regions may either be conserved or significantly diverged. Based on this observation, we defined two types of conserved disorder: 1) “constrained disorder”, regions where the amino acid sequence is well conserved, and 2) “flexible disorder”, regions where the amino acid sequence has diverged. Our analyses revealed that these two types of conserved disorder have different biophysical and biological properties. Flexible disorder is predominantly associated with signaling and regulation, whereas constrained disorder is associated with chaperones and ribosomal proteins.

Here, we investigate the roles of these different forms of disorder in metazoans, with a focus on the human proteome. We provide evidence for distinct roles for disorder in tissue-specific regulation. In particular, we find different roles for constrained and flexible disorder in relation to alternatively spliced regions of proteins, phosphorylation sites and short linear motifs. While flexible disorder may predominantly function by providing structural flexibility that enables the expression and folding of splice isoforms, constrained disorder appears to provide structural scaffolding for presentation of linear motifs and phosphorylation sites, enabling tissue-regulated alternative splicing to rewire signaling pathways and protein interaction networks.

## Results

### A new role for disorder in tissue-specific protein regulation

**Figure 1:**
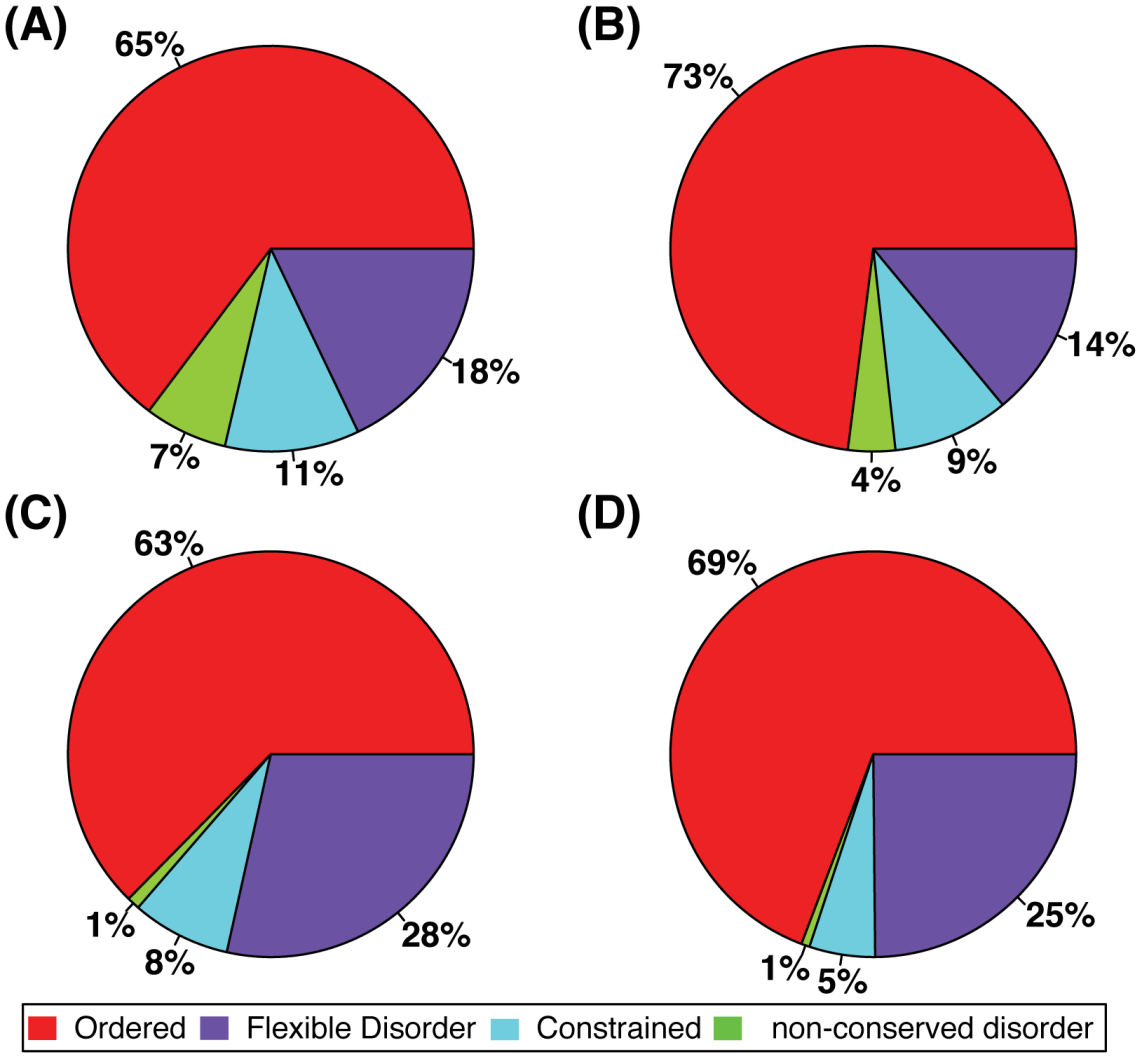
Comparison of disorder rates in the yeast and human proteomes. The relative rates of flexible, constrained and non-conserved disorder in the human proteome are shown. Percentages of the different categories in A) yeast proteins without human orthologs, B) yeast proteins with human orthologs, C) human proteins without yeast orthologs, D) human proteins with yeast orthologs. The human proteome contains higher rates of flexible disorder than the yeast proteome. Proteins without yeast orthologs, which are presumably younger, have higher rates of disorder.

Using our previously described methodology [15], we analyzed the distribution of conserved flexible and constrained disorder in human proteins. To ensure reliable disorder prediction and sequence alignment we used two different and independent strategies, which yielded qualitatively similar results (See Methods and Text S1). As the assignment of the two types of conserved disorder categorization is dependent on the cut-off values used to classify residues as disordered and conserved, we employed steps to ensure consistent criteria in our analyses (See Methods). Specifically, we sought to maximize consistency in assignments of disorder category between the current work and previous study in yeast [15] i.e., residues in human proteins should be assigned the same category as the corresponding residue in their yeast ortholog (if existent). Among all orthologous proteins, we observe 61% overlap between assigned disordered residues in both species. Interestingly, there is a significantly higher overall level of conserved flexible disorder in human compared to yeast proteins (79% vs. 38%; P = 0, Chi-squared Test). In contrast, when comparing human proteins that have yeast orthologs, which are an older evolutionary origin, with human proteins that lack yeast orthologs, there is significantly more constrained disorder in the latter set (5% and 8%, respectively; P = 0, Chi-squared Test). Similarly, yeast proteins that lack human orthologs on average have a slightly higher level of constrained disorder (See Figure 1). It is interesting to consider that the significant increase in constrained disorder in more recently evolved human proteins may be associated with increase in organismal complexity. Likewise, the increase of flexible disorder in such human proteins may be associated with a higher rate of neutral change, which may provide a basis for the evolution of new functions.

To further examine the possible role of conserved constrained and flexible disorder, we performed a functional enrichment analysis of proteins containing relatively high proportions of flexible or constrained disordered residues (See Methods). We find that both flexible and constrained disorder are enriched in proteins with functions related to cell differentiation and development (See Table S1). For example, proteins enriched in flexible disorder are significantly associated with categories such as erythrocyte differentiation and osteoblast development. Likewise, proteins with constrained disorder are enriched in functions associated with fibroblast migration and smooth muscle development. This is consistent with our earlier findings focusing on the yeast clade, in which we found that disorder is closely related to regulatory functions, rather than structural or enzymatic activities. Regulatory function in human proteins is often related to cell differentiation and development and, evidently, disordered regions play an important role in these processes [15].

### Relationships between disorder and alternative splicing

Regulation of tissue-specificity can be achieved through multiple processes including differential gene expression [16], posttranslational modification [17] and alternative splicing [18]–[22]. To better understand the role of conserved disorder in determining tissue-specificity, we explored its relationship with tissue-specific regulation at the levels of mRNA expression, alternative splicing and phosphorylation. We observe that constrained disorder is weakly although significantly correlated with tissue-specificity in mRNA expression (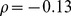, P<2.2e-16, see Methods and Figure S2) [23], [24]. However, we observe a stronger association between constrained disorder and tissue-regulated AS (see below).

**Figure 2:**
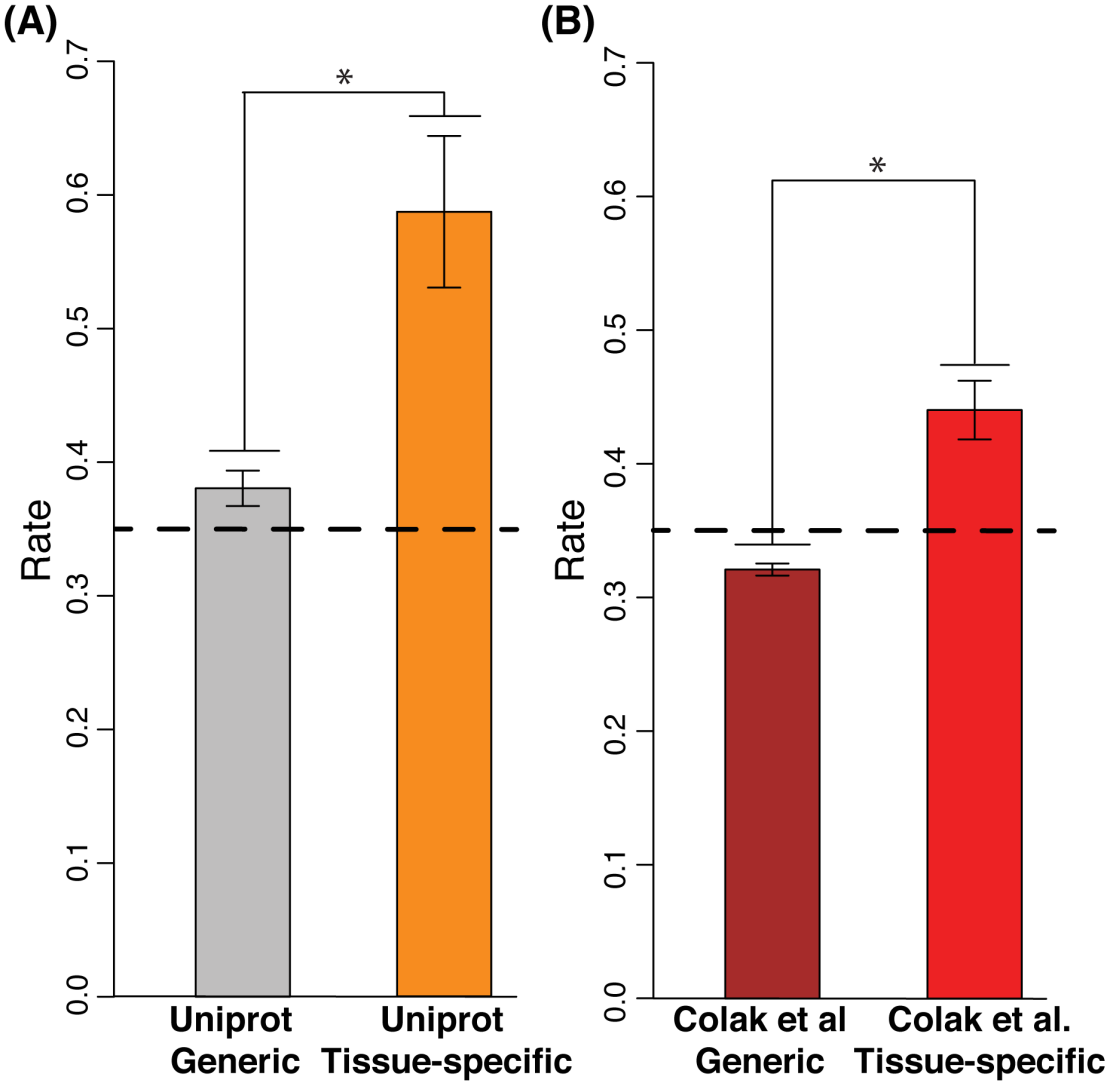
Disorder in alternatively spliced exons. A) The set of exons annotated in UniProt as alternatively spliced can be split into two sets: Bona-fide Tissue-Specific and bona-fide General. We show here that while general alternatively spliced exons are only slightly enriched in disorder, tissue-specific exons are highly enriched in disorder (P<1.7e-5, Wilcoxon rank-sum test). The dotted line refers to the background level of disorder in the proteome. B) Using a larger set of alternatively spliced exons, tissue-specific alternative exons are found to be highly enriched in disorder (P<4.7e-7), whereas general alternative exons are not. The dotted line refers to the background level of disorder in the proteome.

We have recently shown that tissue-specific exons are enriched in regions of highly disordered amino acid sequences, and that these exons often function in controlling PPIs in networks [8]. In contrast to a previous report [6], we found that alternatively spliced exons that are not alternatively spliced in a tissue-specific manner, termed here as general AS events, are not significantly enriched in disordered regions (see also Figure 2A). Here, we resolve this apparent discrepancy. The Romero *et al*. study mostly analyzed UniProt-annotated alternatively spliced exons, which are enriched in tissue-specific AS exons (P<0.004, Chi-squared test, See Text S1). In fact, by pre-defining a bona-fide set of proteins with tissue-specific AS exons, we find that the UniProt set of proteins contain approximately the same level of disorder as our set, whereas exons that are not pre-selected as tissue-specifically regulated in the UniProt set have a markedly lower level of disorder and are very close to the genomic average (See Figure 2B). Our findings underline the importance distinguishing between tissue-specific and general AS exons when establishing relationships between disorder and AS.

**Figure 3:**
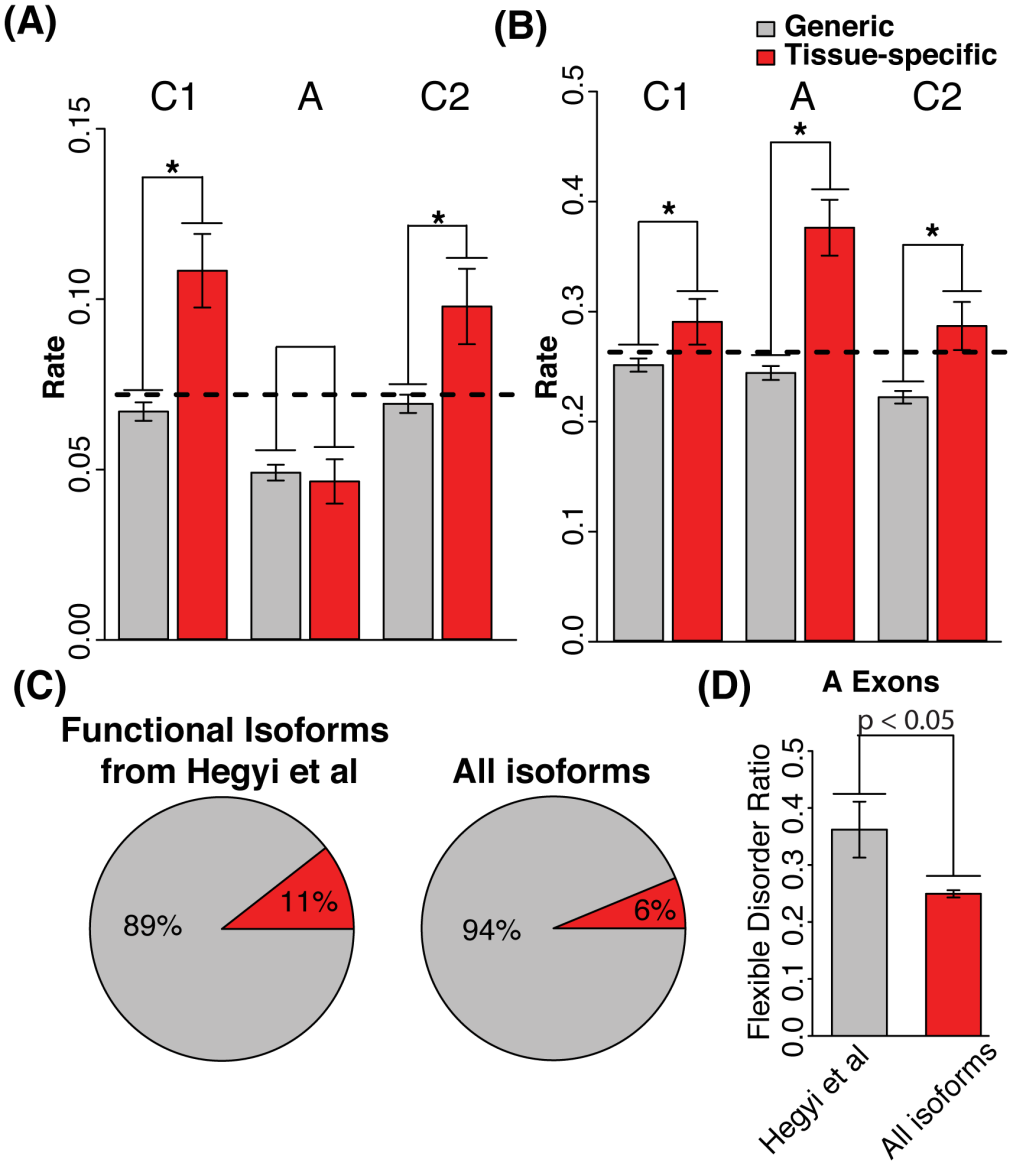
Constrained and flexible disorder in alternatively spliced and flanking exons. A) Constrained disorder is enriched in flanking constitutive (C1 and C2) exons (P<5.64e-7 and P<2.14e-3 respectively, Wilcoxon rank-sum test), whereas tissue-specific alternatively spliced exons (A) are not enriched in constrained disorder. B) Flexible disorder is highly enriched in tissue-specific alternative (A) exons (P<3.36e-5, Wilcoxon rank-sum test). Conversely, in flanking C1 and C2 exons, it is less enriched (P<2.18e-2 for C1 and P<8.45e-3 for C2, Wilcox rank-sum test). C) Functional proteins are enriched in tissue-regulated alternative exons (P<0.03, Wilcoxon rank-sum test). D) AS exons of functional proteins are enriched with flexible disorder compared to AS exons of other proteins. (P<0.05, Wilcoxon rank-sum test).

Importantly, when extending the above analysis by further categorizing conserved protein disorder into subgroups associated with AS regions of proteins, we observe several interesting relationships. While tissue-specific alternative exons have a significantly higher rate of flexible disorder relative to general alternative exons (i.e. those exons that are generally not subject to tissue regulation), conserved constrained disorder is not enriched in these exons (P<3.36e-5 for flexible disorder, Mann-Whitney test; see Figure 3A and Figure 3B). In contrast, the constitutive exons immediately flanking the tissue-specific alternative exons are significantly enriched in both flexible and constrained disorder when compared to general alternatively spliced exons. Similar results are observed when controlling for potential biases stemming from alignment methodology, alignment quality, or from disorder prediction methodology, as well when controlling for possible biases due to alternative exons missing in some orthologs (see Text S1 and Figures S5, S6, S7, S8).

The enrichment in flexible disordered amino acids in tissue-specific alternative exons is consistent with the hypothesis that disordered regions afford structural flexibility such that exons can be alternatively spliced in or out without jeopardizing protein stability [6]. This view is consistent with previous observations that regulated AS events are under-represented in folded domains of proteins [8], [9], [20], [25], [26], while transcripts harboring such AS events appear to be generally translated [27], although in some cases it has been reported that alternatively spliced exons lead to misfolded or unstable proteins, which are degraded [28], [29]. This latter situation may in some cases provide a form of post-translational regulation [29]. Furthermore, a subset of AS events will lead to low-abundance isoforms, including those containing premature termination codons, which are often targeted by nonsense mediated mRNA decay (NMD) and are less likely to be translated [30], [31].

Given these possible scenarios, we determined whether our set of proteins containing tissue-specific alternative exons are enriched in bona-fide proteins listed in Hegyi *et al*. [32] (i.e., proteins for which there is evidence from mass spectroscopy studies), over the set of proteins that contain general alternative exons. Indeed, we find proteins harboring tissue-regulated alternative exons are significantly more often likely to be functional (See Methods), consistent with the idea that tissue-specific AS events affect tissue development and identity through the regulation of protein function (P<0.03, Chi-squared Test, See Figure 3C). Further supporting this conclusion, as found for tissue-regulated alternative exons, we find that alternative exons overlapping bona-fide proteins are also significantly enriched in flexible disorder, compared to the general alternative exons (p<0.05, Mann-Whitney Test, See Figure 3D). These results suggest that the enrichment of tissue-regulated alternative exons in flexible disorder in is largely due to structural reasons, i.e., to aid the folding and stability of both alternative isoforms.

We also observe a second, distinct relationship between conserved disorder and tissue-regulated AS events, namely, that both flexible and constrained disorder are significantly enriched in the constitutive exons immediately flanking the alternatively spliced exons (see Figure 3A and 3B). The majority of interactions in signaling pathways are mediated by short, flexible interfaces that can be detected at the sequence level as linear motifs. These motifs mostly occur in disordered regions due to the conformational flexibility afforded by these regions, which is important for their recognition. Some are bound by peptide binding domains such as SH3 domains, while others are sites of post-translational modification, e.g., by protein kinases. Taken together with our recent results revealing a widespread role for tissue-specific alternative exons in controlling PPIs [8], we considered that the enrichment of the flanking constitutive exons in flexible disorder may be important for controlling interactions mediated by the adjacent alternative exons. Accordingly, we sought to better define the linear motifs and phosphosites associated with alternatively spliced exons.

### Linear motifs and phosphosites are enriched in flanking constitutive exons, but not in alternatively spliced exons

**Figure 4:**
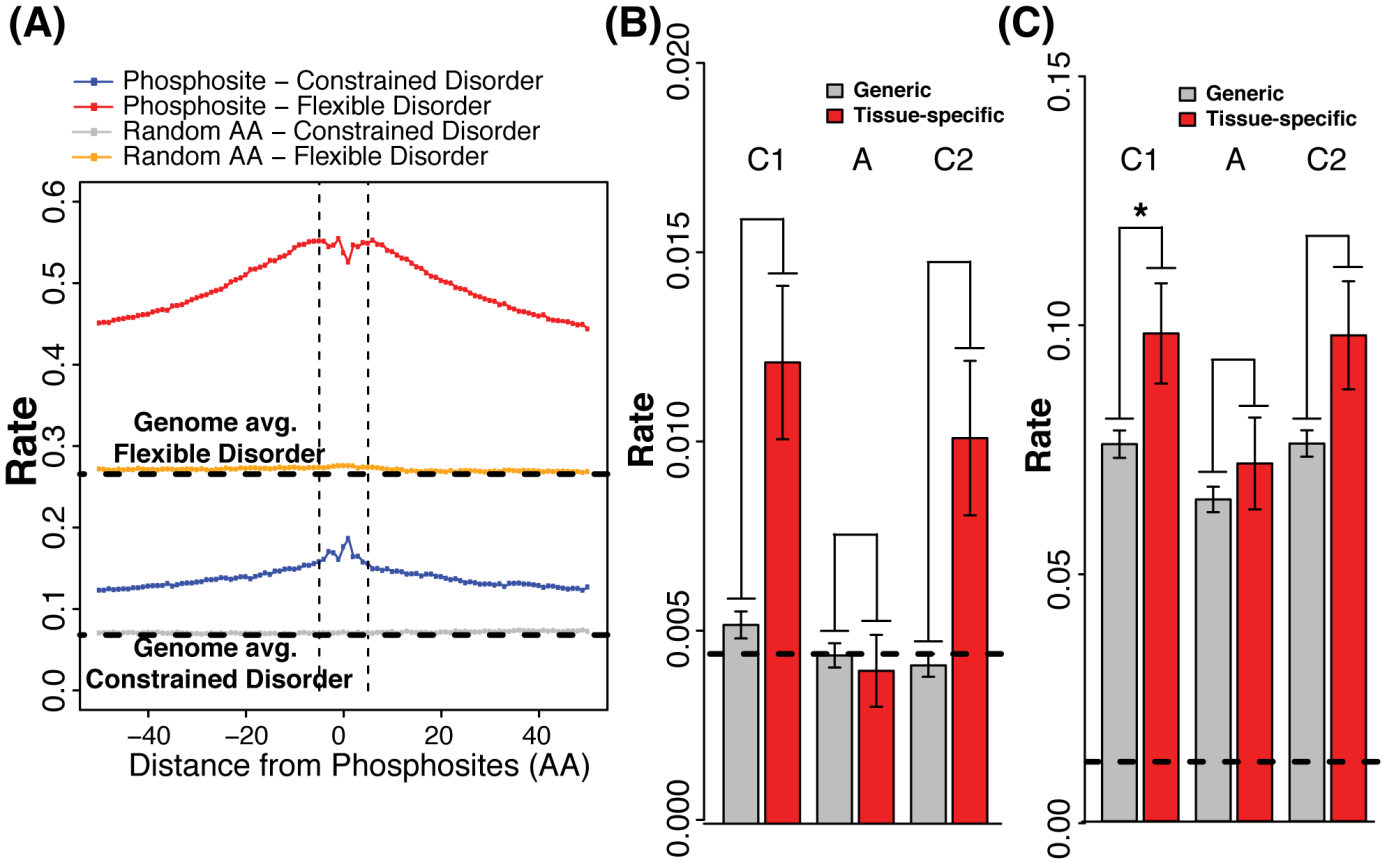
Phosphorylation sites and linear motifs in alternative splicing and disorder. A) Constrained disorder enrichment peaks at the phosphosite position, whereas flexible disorder peaks in the two flanking regions. B) Phosphosites are highly enriched in C1 and C2 exons (P<8.14e-5 and P<6.84e-6 respectively), but not in A exons (P<0.96). C) Linear motifs are enriched in C1 and C2 flanking exons (P<1.47e-3 and P<0.56 respectively), but not in tissue-specific A exons (P<0.22).

First, we analyzed the role of flexible and constrained disorder with respect to phosphosites and linear motifs. Consistent with earlier results, we find that both kinds of disorder are enriched in these protein features [15]. Extending this, we find that while actual phosphosites and linear motifs are associated with a peak in constrained disorder, the immediate flanking regions have comparatively higher rates of flexible disorder (See Figure 4A). This finding leads to one tempting image: regions around phosphosites are enriched in flexible disorder, thereby providing flexibility needed for phosphorylation. Conversely, the phosphosite itself tends to be conserved, rendering it to be more enriched in constrained disorder.

Next, we investigated the extent of enrichment of phosphosites and linear motifs in regions surrounding alternatively spliced exons. Zhang *et al*. previously observed an enrichment of phosphosites in proteins regulated by the Nova splicing factor [33]. While previous studies found enrichment for linear motifs in alternatively spliced exons [7], [9], we find strong enrichment for both features in exons flanking the alternative exon, but no measurable enrichment in the alternative exon itself (See Figure 4B, 4C and also Text S2 for comparison against recent findings of Buljan *et al* [9]). It suggests that the role of disorder in alternative exons likely differs from that in flanking exons. In particular, constitutive exon flanks may provide scaffolding for regulatory roles of linear motifs and phosphosites, while flexible disorder in alternatively spliced exons may largely have a structural role (see above).

### Increases in linear motifs account for enrichment of disorder in regions flanking tissue-regulated alternative exons

**Figure 5:**
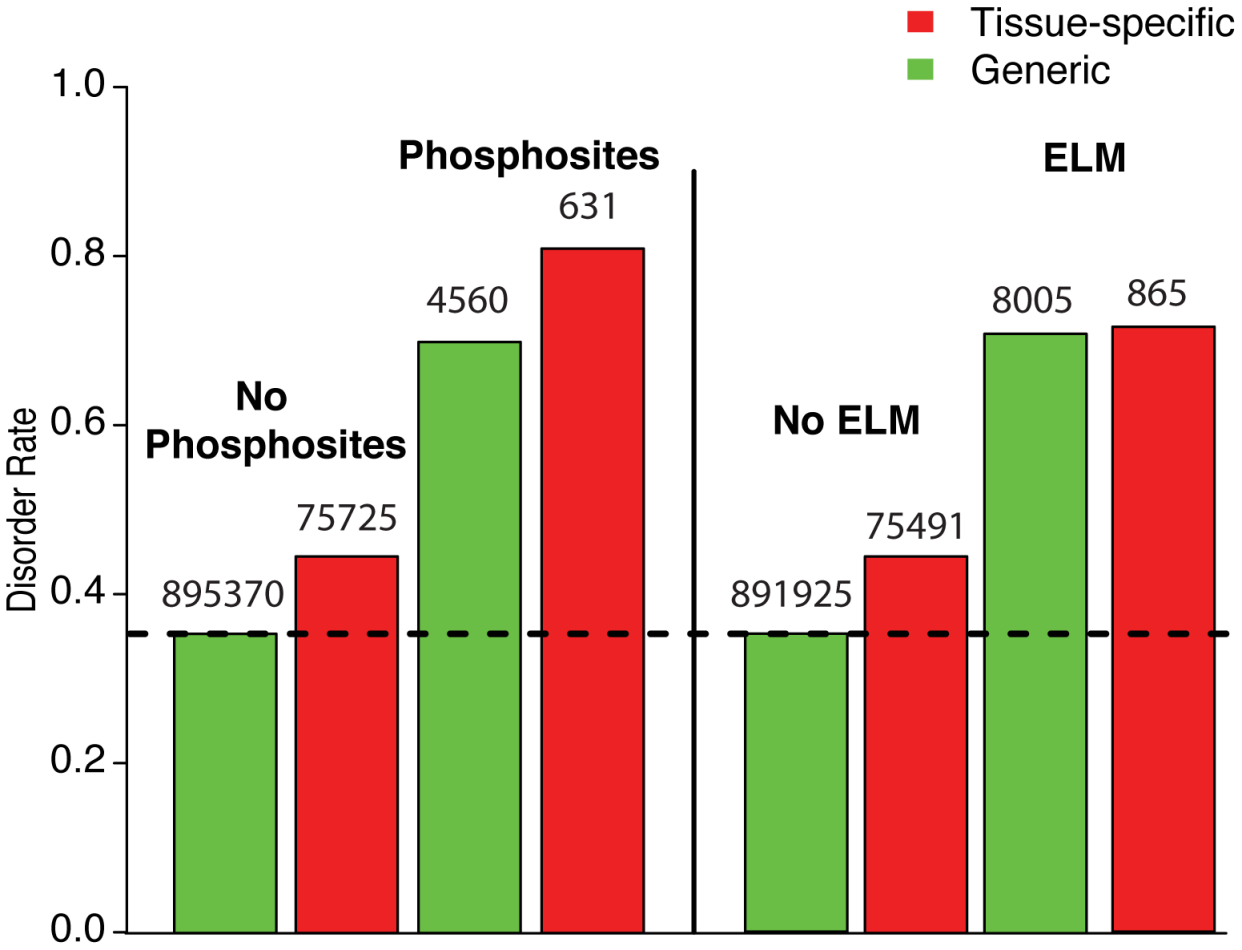
The enrichment of disorder around alternatively spliced exons is driven by phosphosites and ELMs. Disorder rates of residues in different alternatively spliced exons. Left: Disorder rates of residues with and without phosphosites in general alternatively spliced exons and of residues with and without phosphosites in tissue-specific alternatively spliced exons. While the increase in disorder rate is modest between residues in general to tissue-specific exons, a much stronger increase is observed when comparing between residues with and without phosphosites. (All differences are significant with P<1e-16, Wilcoxon rank-sum test). Right: Disorder rates of residues with and without linear motifs in general alternatively spliced exons and of residues with and without linear motifs in tissue-specific alternatively spliced exons. While the increase in disorder rate is modest between residues in general to tissue-specific exons, a much stronger increase is observed when comparing between residues with and without linear motifs. (All differences are significant with P<1e-16, Wilcoxon rank-sum test).

We compared the rates of constrained disorder of residues within and outside of phosphosites and linear motifs, respectively, in constitutive exon flanks and in randomly selected distal exons. In other words, in this analysis we compared the increase in constrained disorder due to the presence of a phosphosite or linear motif to the increase due to tissue-specific alternative splicing. We find that the enrichment for constrained disorder in exons flanking tissue-specific AS exons are to a large extent driven by the presence of phosphosites and linear motifs (Figure 5). In particular, compared to the proteome-wide disorder rate average of 36%, we find that tissue-specific exons outside of phosphosites are slightly enriched in disorder (45%), while a larger increase in enrichment of both constrained and flexible disorder is observed for residues located around phosphosites and ELMs (81%). Interestingly, when performing the same analysis for alternative exons and flexible disorder, we observe a relatively large enrichment for flexible disorder (>52% See Figure S3) that is independent of phosphosites or ELMs compared to the proteome-wide average of 20%. This observation is consistent with our earlier result that the enrichment of flexible disorder in tissue-specific alternative exons is due to structural flexibility.

### Alternatively spliced exons and their flanking exons are enriched in cancer driver mutations

Both disordered regions and linear motifs are known to have important roles in regulation of many cellular processes and have been implicated in numerous diseases. As we observed significant enrichment of flexible and constrained disorder in tissue regulated exons and flanking exons, respectively, we therefore next asked whether such regions are associated with disease mutations. More specifically, we asked whether mutations implicated in driving cancer growth are enriched in these regulation “hot spots”. For control and comparison purposes, we investigated enrichment of cancer mutations in general alternative exons and flanking exons. Abnormal perturbations in cell regulation due to genetic mutations can result in uncontrolled cell proliferation and tumor formation [34]. Such changes are caused by “driver” mutations, i.e., mutations that provide a growth advantage. By contrast, the majority of somatic mutations in cancer are “passenger” mutations that accumulate in the cancer genome as a result of a breakdown of DNA repair processes [35]. To define driver and passenger mutations, we used cancer mutation frequency information from the Catalogue of Somatic Mutations in Cancer (COSMIC) [36], [37]. For our analysis, we classified driver mutations based on their occurrence in multiple independent tumor samples, whereas passenger mutations were present in single tumor samples (See Methods for details).

**Figure 6:**
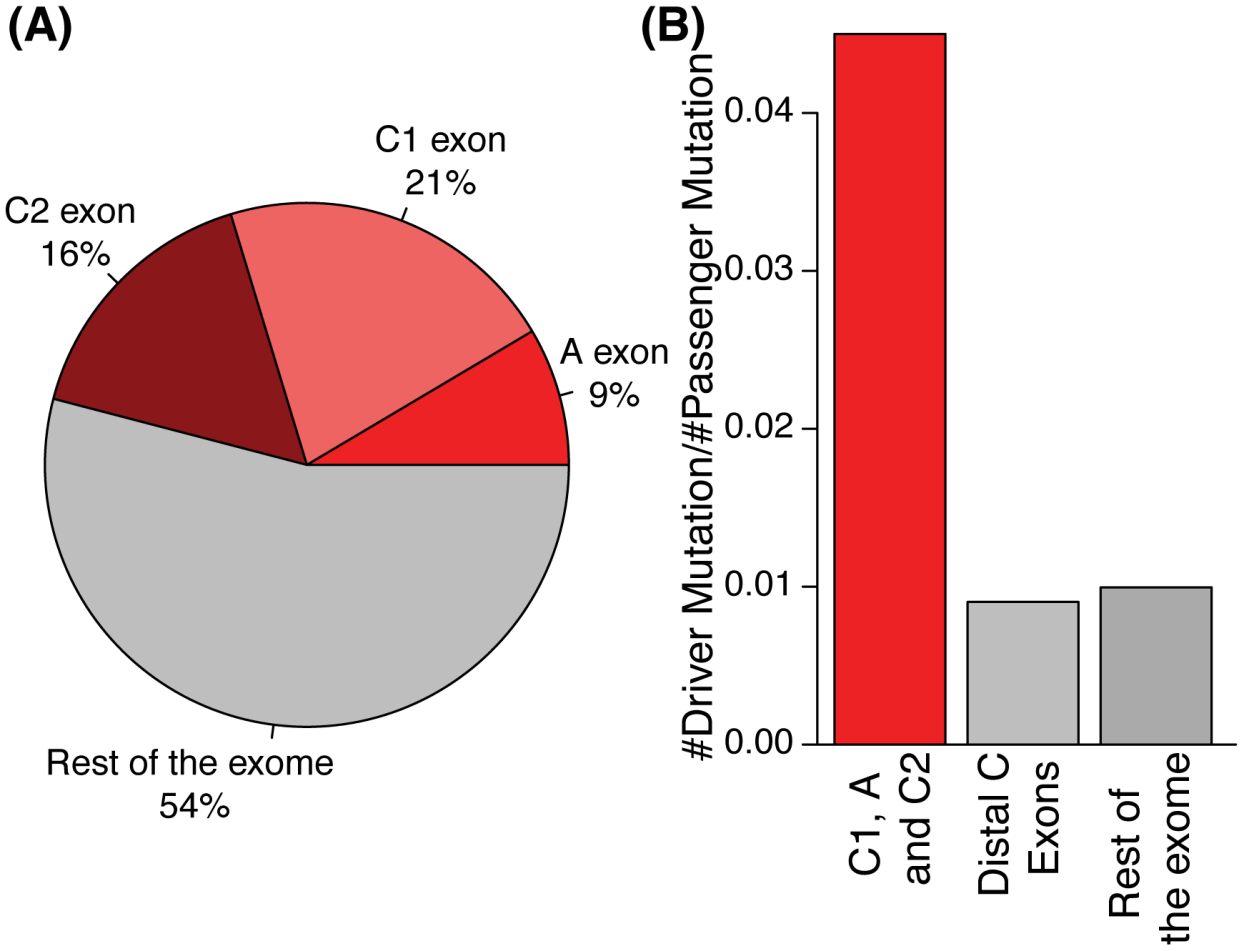
Enrichment of driver cancer mutations in alternatively spliced regions. A) Percentage of driver mutations that lie in different types of exons. A significant fraction of driver mutations falls within A, C1 and C2 exons. B) Ratio of driver to passenger mutations in different types of exons (A, C1 and C2 exons, distal C exons and rest of the exome). A significantly higher ratio of driver to passenger mutations is observed in A, C1 and C2 exons.

Although we did not observe significant enrichment of driver mutations in regions containing tissue specific AS events compared to regions containing general AS events, we did observe an overall significant enrichment of driver mutations in AS neighborhoods (Figure 6A) compared to randomly selected exons. Remarkably, 690 of 1502 (46%) driver mutations were detected in alternative splicing regions encompassing alternative (A) exons and flanking constitutive exons (C1 and C2). Specifically, there is a density of 0.43, 0.93 and 0.49 driver mutations per 10 Kb in C1, A and C2, respectively, whereas the density in the overall exome is 0.24 driver mutations per 10 Kb. Since the A and flanking C1 and C2 exons constitute only a small portion of the coding genome (∼10 million nucleotides as per our dataset), this enrichment is highly significant as revealed by a Chi-square test (P<1.99e-108), when comparing the ratios of driver vs. passenger mutations in alternative splicing neighborhoods as compared to the rest of the exome. Our results remain qualitatively unchanged when we use other frequency thresholds for calling driver and passenger mutations, indicating robustness of our observations (See Methods). Moreover, a missense mutation occurring in an alternatively spliced neighborhood is ∼5 times more likely to be a driver than a passenger mutations when compared to constitutive distal exons in the same proteins (See Figure 6B, P<2.59e-63, Chi-square Test). Likewise, it is more than 4.5 times more likely to be a driver than a passenger mutation compared to mutations occurring in the rest of the exome (P<5.9e-202, Chi-square Test).

These results provide evidence that alternatively spliced exons and their flanking exons are hot spots for cancer driver mutations. Although we did not observe significant enrichment of driver or passenger mutations in tissue-regulated exons or their flanking constitutive exons, driver mutations were nevertheless detected in these regions. Given the importance of these regions in the regulation of protein-protein interactions and in signaling, it is therefore important to consider that such disease mutations in these regions may result in the rewiring of signaling and protein-protein interaction networks in cancer cells. Conversely the enrichment of driver mutations in regions that are alternatively spliced but not annotated as undergoing tissue regulation could reflect possible selection acting to avoid disruption of regions of proteins that are more often associated with formation of interaction hubs in protein interaction networks. Conversely, it is also possible that many such regions annotated as being “general” AS, are in fact regulated in a tissue-specific or condition-specific manner but were not detected as such using the limited panel of RNA-Seq data employed in this study. Regardless, these results provide a basis for future investigations addressing the mechanisms by which cancer driver mutations contribute to the onset and progression of tumors.

## Discussion

In this work we used a comparative proteomics approach to investigate fundamental properties of conserved disorder in higher eukaryotes. Our results suggest that conserved flexible disorder may largely have a structural role associated with tissue-specific alternative splicing, whereas conserved constrained disorder has a regulatory role by providing scaffolding for linear motifs. As it becomes increasingly evident that alternative splicing affects a substantial fraction of the proteome and is an important determinant in controlling protein interactions, future studies will be facilitated by taking these different possible roles of disorder into account. It will be of considerable interest to determine the different functional relationships between AS and the various protein motifs and features that we find are enriched in and proximal to tissue-regulated alternative exons in this study. In particular, it will be important to address the role of specific arrangements of linear motifs in the regulation of protein-protein interactions [8]–[10]. The lack of enrichment of interaction motifs in regulated alternative exons may imply that these exons attenuate interactions that are mediated by linear motifs or phosphosites in flanking constitutive exons (where they are enriched). On the other hand, the alternatively spliced exon may represent the main site of the protein-protein interaction and its affinity may be modulated by the modification status of sites within the flanking exon regions, with the interaction dependent on both splicing and phosphosite or the status of other PTMs. Our results thus provide interesting testable hypotheses that can be addressed in future experiments. Finally, we provide new insight into relationships between cancer driver mutations, AS, and protein composition and function, that will facilitate future studies directed at determining mechanisms underlying the growth and spread of cancer cells.

## Methods

### Orthologue selection and alignment

The selection of human proteins were made from 81968 human proteins in *Ensembl* (v57.0) [38] using two rules:

1. The protein identifier mapped to CCDS [39].
2. The protein had more than 15 orthologues within the Eukaryotes [40].

In the event of one-to-many and many-to-many ortholgous relationships for a given human protein, blastp was used to select the closest orthologue by using the lowest e-value. The resulting 28781 orthologue groups spanning 51 eukaryote species were aligned using the multiple sequence alignment tool MAFFT with default options [41], [42]. 22 of 55 species were selected to be sufficiently diverse in order to prevent the over estimation of sequence conservation [43], [44] (See Figure S4). To avoid biases due to the alignment tool, we also used an alternate alignment strategy (See Text S1).

### Protein disorder

Protein disorder was derived using the software Disopred2 with default settings [45]. To avoid biases due to the disorder prediction algorithm, we also used an alternate prediction tool (See Text S1).

### Calculation of residue and disorder conservation score

Amino acid conservation and disorder conservation scores were calculated in the same manner as in Bellay *et al* [46]:

Amino acid conservation score (**A_n_**) of position **n** in an alignment with **K** sequences is calculated and binned as follows:
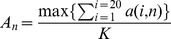
Where **a(i,n)** is the number of sequences that has amino acid of type **i** on position **n**. Next we binned each position as follows:
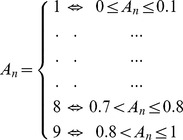
The disorder conservation score (**D_n_**) is the binned score (the same conservation binned scoring scheme) of the percentage of species in a multiple sequence alignment retaining the same disorder classification. This is achieved by superimposing the disorder classification for each amino acid by *Disopred2* [45] on the previously described multiple sequence alignment.

### A systematic classification of disorder

Conserved disorder refers to aligned positions that have D> = 3, indicating that > = 30% of aligned residues are disordered. This category contains two classes:

1. Constrained disorder: aligned positions where D> = 3 and A> = 9, indicating that the selected sequences are disordered in 30% or more of aligned residues and conserved in 80% or more of aligned residues.
2. Flexible disorder: aligned positions where D> = 3 and A<9, indicating that the selected sequences are disordered in 30% or more of aligned residues and conserved in less than 80% of the aligned residues.

### GO enrichments

GO term enrichment for each class (constrained and flexible disorder) was performed by binning into one of the categories classes based on its maximum proportion of residues in that class. The distribution of disorder for each GO term was tested against the background distribution of that disorder type using the Wilcoxon Rank Sum test for p-value<0.05, where p-value was adjusted for multiple hypotheses testing using false discovery rate.

### Tissue-specificity and gene expression

We used the RNA-Seq data from Illumina's Human BodyMap 2.0 project, which was kindly provided by Dr. Gary Schroth (Illumina) and recently documented by Rinn and colleagues [47]. The data consist of 16 human tissue types, including adrenal, adipose, brain, breast, colon, heart, kidney, liver, lung, lymph, ovary, prostate, skeletal muscle, testes, thyroid, and white blood cells. We trimmed all reads to 50 nucleotides, and used only the forward end. We then mapped the reads to the transcriptome using bowtie [48] with –m 1 –v 2 parameters (requiring unique mapping and two or less mismatches across the full alignment). We performed multiple mapping corrections as follows: each position in each transcript using 50-nt windows was mapped back against the whole transcriptome. If the sequence mapped somewhere else in addition to itself we discard it and discounted from the transcript effective length (length-49). We then used the “effective length” to divide the raw read counts per million mapped reads for each gene to obtain corrected-RPKM values (cRPKM). We then used a conservative cRPKM cutoff of 10, and called a gene expressed in a given tissue if cRPKM> = 10. Finally, we derived a tissue-specificity score for each of the 17039 genes as follows:
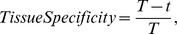
where ***t*** is the number of tissues the genes is expressed in and T = 16 is the total number of tissues considered.

### Alternative splicing

Using the same RNA-Seq dataset described above in addition to the alternative splicing events previously mined (See [8] for details) from the BodyMap dataset. Of the 27,240 distinct human cassette exon alternative splicing (AS) events from RNA-Seq data, 16050 of these events were mapped to the subset of Ensembl protein isoforms (explained above) with high confidence. Of these, we used only the 4328 AS events that had both the inclusion and exclusion isoforms mapped. We refer to this dataset as the ‘general AS’ event set. From this set, we further derived a set of 268 tissue-specific events that we previously called as specific to one or more of the tissues listed above. See Supplementary material in [8] for detailed description of categorization of alternative splicing events into constitutive, general and tissue-specific events.

### Phosphorylation sites and Eukaryotic Linear motif sites

Human phosphorylation sites were obtained from PhosphoSitePlus [49] and Phospho.ELM [50]. We used 77615 phosphorylation sites from 13010 proteins. ELM sites were kindly provided by Dr. Norman Davey (EMBL, Heidelberg) who used SLiMSearch 2.0 [51] tool to generate the high-quality ELM dataset.

### Enrichment map

We used Cytoscape [52] and the Enrichment Map plugin [53] to create the Enrichment Maps. The edges represent the value of the overlap coefficient (size of the intersection of both GO terms/size of the small GO term) with a cutoff at 0.4.

### Cancer mutations

The mutation data was obtained from the Sanger Institute Catalogue Of Somatic Mutations In Cancer web site, http://www.sanger.ac.uk/cosmic [36].

Somatic missense mutations from 98463 amino acid sites were downloaded (version 59). Classification of driver mutation sites and passenger mutation sites were determined by their mutation frequency. Missense mutations were defined as a driver mutation if at least 5 distinct COSMIC samples from at least 3 distinct studies. To prevent bias from low throughput, targeted gene analysis, we also called mutations coming from in at least 3 distinct samples from whole genome screening based studies as driver mutations. We obtained 1502 driver and 97961 passenger mutations. While the frequency thresholds used were arbitrary set due to lack of a golden truth set, we observed that our results remain qualitatively unchanged even when using a range of thresholds for calling driver and passenger mutations, implying robustness of our observations.

## Supporting Information

Figure S1 Each network is a representation of the GO terms over-represented in the sets of proteins enriched in (A) Constrained disorder, (B) Flexible disorder. Each node represents a GO terms, its size indicating the significance of the enrichment (the bigger the node, the more significant the enrichment). Edges represent overlap between two GO terms (Overlap coefficient).
(TIF)

Figure S2 The boxplots show the correlation between the tissue specificity of the gene and the portion of (A) flexible disorder and (B) constrained disorder. All genes are binned into 5 different bins depending on the tissue specificity score.
(TIF)

Figure S3 The enrichment of disorder, constrained disorder, and flexible disorder in different types of exons is largely driven by phosphosites and ELMs. (A–C) C1 exons, (D–F) A exons, (G–I) C2 exons.
(TIF)

Figure S4 The species chosen for analyses are labeled red in the phylogenetic tree.
(TIF)

Figure S5 Ratio of gaps for each region types based on the orthologs alignments generated by (A) MAFFT and the (B) the MUSCLE multiple sequence aligners. Gap rate is calculated as average gap ratio within the exon/region, which is calculated as the number of gaps for a given site divided by number of species in the alignment.
(TIF)

Figure S6 Conserved disorder rate analysis using the MUSCLE and IUPred tool combination. (A) Constrained disorder is only enriched in flanking (C1 and C2) exons (P<3.62e-08 for C1 and P<0.0003 for C2). The tissue-specific alternatively spliced exons are not enriched in constrained disorder. (B) Flexible disorder is highly enriched in tissue-specific A exons (P<6.91e-08).
(TIF)

Figure S7 Analysis of effect of systematic removal of gapped regions from (A) MAFTT and DisoPred2 based flexible disorder rate analysis (B) from MUSCLE and IUPred based flexible disorder rate analysis (C) from MAFTT and DisoPred2 based constrained disorder rate analysis (D) from MUSCLE and IUPred based constrained disorder rate analysis reveal no elevated rates of exon content difference within orthologs of tissue specific A exon containing isoforms compared to exon content difference in orthologs of general A exon containing isoforms.
(TIF)

Figure S8 (A)–(J): Visualization of A exon regions of MAFTT orthologs alignments of randomly selected 10 tissue specific, highly flexible (>0.8) A exons reveals no systematic exon content difference.
(TIF)

Table S1 The Gene Ontology (GO) along with respective enrichment p-values, for proteins of high content of flexible and constrained disorder. The protein is classified as either constrained disorder or flexible disorder if constrained (or flexible) disorder is the dominating class among 4 different classes: constrained disorder, flexible disorder, ordered, and non-conserved.
(XLS)

Text S1 Alternative alignments and disorder prediction methodology. Results obtained from re-implementing our pipeline with MUSCLE [42] and IUPred [54] tool combination.
(DOCX)

Text S2 A note on results of Buljan *et al* [9]. Comparison of our ELM enrichment against the results reported in Buljan *et al* [9].
(DOCX)

## References

[1] Dyson HJ, Wright PE (2005) Intrinsically unstructured proteins and their functions. Nat Rev Mol Cell Biol 6: 197–208. 15738986 10.1038/nrm1589

[2] Ward JJ, Sodhi JS, McGuffin LJ, Buxton BF, Jones DT (2004) Prediction and functional analysis of native disorder in proteins from the three kingdoms of life. J Mol Biol 337: 635–645 doi:10.1016/j.jmb.2004.02.002. 15019783 10.1016/j.jmb.2004.02.002

[3] Pentony MM, Jones DT (2010) Modularity of intrinsic disorder in the human proteome. Proteins 78: 212–221. 19626706 10.1002/prot.22504

[4] Gsponer J, Futschik ME, Teichmann SA, Babu MM (2008) Tight regulation of unstructured proteins: from transcript synthesis to protein degradation. Science 322: 1365–1368 doi:10.1126/science.1163581. 19039133 10.1126/science.1163581 PMC2803065

[5] Lobley A, Swindells MB, Orengo CA, Jones DT (2007) Inferring function using patterns of native disorder in proteins. PLoS Comput Biol 3: e162. 17722973 10.1371/journal.pcbi.0030162 PMC1950950

[6] Romero PR, Zaidi S, Fang YY, Uversky VN, Radivojac P, et al (2006) Alternative splicing in concert with protein intrinsic disorder enables increased functional diversity in multicellular organisms. Proceedings of the National Academy of Sciences 103: 8390–8395. 10.1073/pnas.0507916103 PMC1482503 16717195

[7] Weatheritt RJ, Luck K, Petsalaki E, Davey NE, Gibson TJ (2012) The identification of short linear motif-mediated interfaces within the human interactome. Bioinformatics 28: 976–982 doi:10.1093/bioinformatics/bts072. 22328783 10.1093/bioinformatics/bts072 PMC3315716

[8] Ellis JD, Barrios-Rodiles M, Colak R, Irimia M, Kim T, et al (2012) Tissue-Specific Alternative Splicing Remodels Protein-Protein Interaction Networks. Molecular Cell 46: 884–892 doi:10.1016/j.molcel.2012.05.037. 22749401 10.1016/j.molcel.2012.05.037

[9] Buljan M, Chalancon G, Eustermann S, Wagner GP, Fuxreiter M, et al (2012) Tissue-Specific Splicing of Disordered Segments that Embed Binding Motifs Rewires Protein Interaction Networks. Molecular Cell 46: 871–883 doi:10.1016/j.molcel.2012.05.039. 22749400 10.1016/j.molcel.2012.05.039 PMC3437557

[10] Davis MJ, Shin CJ, Jing N, Ragan MA (2012) Rewiring the dynamic interactome. Mol Biosyst 8: 2054–2066 doi:10.1039/c2mb25050k. 22729145 10.1039/c2mb25050k

[11] Tompa P, Fuxreiter M, Oldfield CJ, Simon I, Dunker AK, et al (2009) Close encounters of the third kind: disordered domains and the interactions of proteins. Bioessays 31: 328–335. 19260013 10.1002/bies.200800151

[12] Bellay J, Michaut M, Kim T, Han S, Colak R, et al (2012) An omics perspective of protein disorder. Mol Biosyst 8: 185–193 doi:10.1039/c1mb05235g. 22101230 10.1039/c1mb05235g

[13] Uversky VN, Oldfield CJ, Midic U, Xie H, Xue B, et al (2009) Unfoldomics of human diseases: linking protein intrinsic disorder with diseases. BMC Genomics 10 (Suppl 1) S7. 10.1186/1471-2164-10-S1-S7 PMC2709268 19594884

[14] Goh KI, Cusick ME, Valle D, Childs B (2007) The human disease network. Proceedings of the National Academy of Sciences 104: 8685–8690 doi:10.1073/pnas.0701361104. 10.1073/pnas.0701361104 PMC1885563 17502601

[15] Bellay J, Han S, Michaut M, Kim T, Costanzo M, et al (2011) Bringing order to protein disorder through comparative genomics and genetic interactions. Genome Biol 12: R14 doi:10.1186/gb-2011-12-2-r14. 21324131 10.1186/gb-2011-12-2-r14 PMC3188796

[16] Wu C, Orozco C, Boyer J, Leglise M, Goodale J, et al (2009) BioGPS: an extensible and customizable portal for querying and organizing gene annotation resources. Genome Biol 10: R130. 19919682 10.1186/gb-2009-10-11-r130 PMC3091323

[17] Huttlin EL, Jedrychowski MP, Elias JE, Goswami T, Rad R, et al (2010) A tissue-specific atlas of mouse protein phosphorylation and expression. Cell 143: 1174–1189. 21183079 10.1016/j.cell.2010.12.001 PMC3035969

[18] Licatalosi DD, Darnell RB (2010) RNA processing and its regulation: global insights into biological networks. Nature Publishing Group 11: 75–87 doi:10.1038/nrg2673. 10.1038/nrg2673 PMC3229837 20019688

[19] Kalsotra A, Cooper TA (2011) Functional consequences of developmentally regulated alternative splicing. Nature Publishing Group 12: 715–729 doi:10.1038/nrg3052. 10.1038/nrg3052 PMC3321218 21921927

[20] Wang ET, Sandberg R, Luo S, Khrebtukova I, Zhang L, et al (2008) Alternative isoform regulation in human tissue transcriptomes. Nature 456: 470–476 doi:10.1038/nature07509. 18978772 10.1038/nature07509 PMC2593745

[21] Ule J, Ule A, Spencer J, Williams A, Hu J-S, et al (2005) Nova regulates brain-specific splicing to shape the synapse. Nat Genet 37: 844–852 doi:10.1038/ng1610. 16041372 10.1038/ng1610

[22] Barash Y, Calarco JA, Gao W, Pan Q, Wang X, et al (2010) Deciphering the splicing code. Nature 465: 53–59. 20445623 10.1038/nature09000

[23] Wu C, Orozco C, Boyer J, Leglise M, Goodale J, et al (2011) BioGPS: an extensible and customizable portal for querying and organizing gene annotation resources. Genome Biol 12: R130. 10.1186/gb-2009-10-11-r130 PMC3091323 19919682

[24] Babu MM, van der Lee R, de Groot NS, Gsponer J (2011) Intrinsically disordered proteins: regulation and disease. Current opinion in structural biology 21: 432–440. 21514144 10.1016/j.sbi.2011.03.011

[25] Resch A, Xing Y, Modrek B, Gorlick M, Riley R, et al (2004) Assessing the impact of alternative splicing on domain interactions in the human proteome. J Proteome Res 3: 76–83. 14998166 10.1021/pr034064v

[26] Kriventseva EV, Koch I, Apweiler R, Vingron M, Bork P, et al (2003) Increase of functional diversity by alternative splicing. Trends in Genetics 19: 124–128 doi:10.1016/S0168-9525(03)00023-4. 12615003 10.1016/S0168-9525(03)00023-4

[27] Tress ML, Wesselink J-J, Frankish A, López G, Goldman N, et al (2008) Determination and validation of principal gene products. Bioinformatics 24: 11–17 doi:10.1093/bioinformatics/btm547. 18006548 10.1093/bioinformatics/btm547 PMC2734078

[28] Calarco JA, Xing Y, Caceres M, Calarco JP, Xiao X, et al (2007) Global analysis of alternative splicing differences between humans and chimpanzees. Genes Dev 21: 2963–2975 doi:10.1101/gad.1606907. 17978102 10.1101/gad.1606907 PMC2049197

[29] Misquitta-Ali CM, Cheng E, O'Hanlon D, Liu N, McGlade CJ, et al (2011) Global profiling and molecular characterization of alternative splicing events misregulated in lung cancer. Mol Cell Biol 31: 138–150 doi:10.1128/MCB.00709-10. 21041478 10.1128/MCB.00709-10 PMC3019846

[30] Pan Q, Saltzman AL, Kim YK, Misquitta C, Shai O, et al (2006) Quantitative microarray profiling provides evidence against widespread coupling of alternative splicing with nonsense-mediated mRNA decay to control gene expression. Genes Dev 20: 153–158 doi:10.1101/gad.1382806. 16418482 10.1101/gad.1382806 PMC1356107

[31] Melamud E, Moult J (2009) Structural implication of splicing stochastics. Nucleic acids research 37: 4862–4872 doi:10.1093/nar/gkp444. 19528068 10.1093/nar/gkp444 PMC2724273

[32] Hegyi H, Kalmar L, Horvath T, Tompa P (2011) Verification of alternative splicing variants based on domain integrity, truncation length and intrinsic protein disorder. Nucleic acids research 39: 1208–1219. 20972208 10.1093/nar/gkq843 PMC3045584

[33] Zhang C, Frias MA, Mele A, Ruggiu M, Eom T, et al (2010) Integrative modeling defines the Nova splicing-regulatory network and its combinatorial controls. Science 329: 439–443 doi:10.1126/science.1191150. 20558669 10.1126/science.1191150 PMC3412410

[34] Blume-Jensen P, Hunter T (2001) Oncogenic kinase signalling. Nature 411: 355–65. 11357143 10.1038/35077225

[35] Stratton MR, Campbell PJ, Futreal PA (2009) The cancer genome. Nature 458: 719–724 doi:10.1038/nature07943. 19360079 10.1038/nature07943 PMC2821689

[36] Bamford S, Dawson E, Forbes S, Clements J, Pettett R, et al (2004) The COSMIC (Catalogue of Somatic Mutations in Cancer) database and website. Br J Cancer 91: 355–358. 15188009 10.1038/sj.bjc.6601894 PMC2409828

[37] Torkamani A, Verkhivker G, Schork NJ (2009) Cancer driver mutations in protein kinase genes. Cancer Lett 281: 117–127. 19081671 10.1016/j.canlet.2008.11.008 PMC2905872

[38] Flicek P, Amode MR, Barrell D, Beal K, Brent S, et al (2011) Ensembl 2011. Nucleic Acids Res 39: D800–D806. 21045057 10.1093/nar/gkq1064 PMC3013672

[39] Pruitt KD, Harrow J, Harte RA, Wallin C, Diekhans M, et al (2009) The consensus coding sequence (CCDS) project: Identifying a common protein-coding gene set for the human and mouse genomes. Genome Res 19: 1316–1323. 19498102 10.1101/gr.080531.108 PMC2704439

[40] Smedley D, Haider S, Ballester B, Holland R, London D, et al (2009) BioMart–biological queries made easy. BMC Genomics 10: 22. 19144180 10.1186/1471-2164-10-22 PMC2649164

[41] Katoh K, Toh H (2010) Parallelization of the MAFFT multiple sequence alignment program. Bioinformatics 26: 1899–1900. 20427515 10.1093/bioinformatics/btq224 PMC2905546

[42] Edgar RC (2004) MUSCLE: multiple sequence alignment with high accuracy and high throughput. Nucleic acids research 32: 1792–1797 doi:10.1093/nar/gkh340. 15034147 10.1093/nar/gkh340 PMC390337

[43] Vilella AJ, Severin J, Ureta-Vidal A, Heng L, Durbin R, et al (2009) EnsemblCompara GeneTrees: Complete, duplication-aware phylogenetic trees in vertebrates. Genome Research 19: 327–335 doi:10.1101/gr.073585.107. 19029536 10.1101/gr.073585.107 PMC2652215

[44] Huson DH, Richter DC, Rausch C, Dezulian T, Franz M, et al (2007) Dendroscope: An interactive viewer for large phylogenetic trees. BMC Bioinformatics 8: 460 doi:10.1186/1471-2105-8-460. 18034891 10.1186/1471-2105-8-460 PMC2216043

[45] Ward JJ, McGuffin LJ, Bryson K, Buxton BF, Jones DT (2004) The DISOPRED server for the prediction of protein disorder. Bioinformatics 20: 2138–2139 doi:10.1093/bioinformatics/bth195. 15044227 10.1093/bioinformatics/bth195

[46] Bellay J, Han S, Michaut M, Kim T, Costanzo M, et al (2011) Bringing order to protein disorder through comparative genomics and genetic interactions. Genome Biol 12: R14. 21324131 10.1186/gb-2011-12-2-r14 PMC3188796

[47] Cabili MN, Trapnell C, Goff L, Koziol M, Tazon-Vega B, et al (2011) Integrative annotation of human large intergenic noncoding RNAs reveals global properties and specific subclasses. Genes Dev 25: 1915–1927 doi:10.1101/gad.17446611. 21890647 10.1101/gad.17446611 PMC3185964

[48] Langmead B, Trapnell C, Pop M, Salzberg SL (2009) Ultrafast and memory-efficient alignment of short DNA sequences to the human genome. Genome Biol 10: R25 doi:10.1186/gb-2009-10-3-r25. 19261174 10.1186/gb-2009-10-3-r25 PMC2690996

[49] Hornbeck PV, Chabra I, Kornhauser JM, Skrzypek E, Zhang B (2004) PhosphoSite: A bioinformatics resource dedicated to physiological protein phosphorylation. Proteomics 4: 1551–1561. 15174125 10.1002/pmic.200300772

[50] Dinkel H, Chica C, Via A, Gould CM, Jensen LJ, et al (2011) Phospho.ELM: a database of phosphorylation sites–update 2011. Nucleic acids research 39: D261–D267 doi:10.1093/nar/gkq1104. 21062810 10.1093/nar/gkq1104 PMC3013696

[51] Davey NE, Haslam NJ, Shields DC, Edwards RJ (2011) SLiMSearch 2.0: biological context for short linear motifs in proteins. Nucleic acids research 39: W56–W60 doi:10.1093/nar/gkr402. 21622654 10.1093/nar/gkr402 PMC3125787

[52] Shannon P, Markiel A, Ozier O, Baliga NS, Wang JT, et al (2003) Cytoscape: a software environment for integrated models of biomolecular interaction networks. Genome Res 13: 2498–2504. 14597658 10.1101/gr.1239303 PMC403769

[53] Merico D, Isserlin R, Stueker O, Emili A, Bader GD (2010) Enrichment map: a network-based method for gene-set enrichment visualization and interpretation. PLoS ONE 5: e13984. 21085593 10.1371/journal.pone.0013984 PMC2981572

[54] Dosztányi Z, Csizmok V, Tompa P, Simon I (2005) IUPred: web server for the prediction of intrinsically unstructured regions of proteins based on estimated energy content. Bioinformatics 21: 3433–4. 15955779 10.1093/bioinformatics/bti541

