# Supplementary figures and images for: Distinct Types of Disorder in the Human Proteome: Functional Implications for Alternative Splicing
Source: PLoS Comput Biol. 2013 Apr 25;9(4):e1003030. doi: 10.1371/journal.pcbi.1003030 (PMC3635989; doi:10.1371/journal.pcbi.1003030)

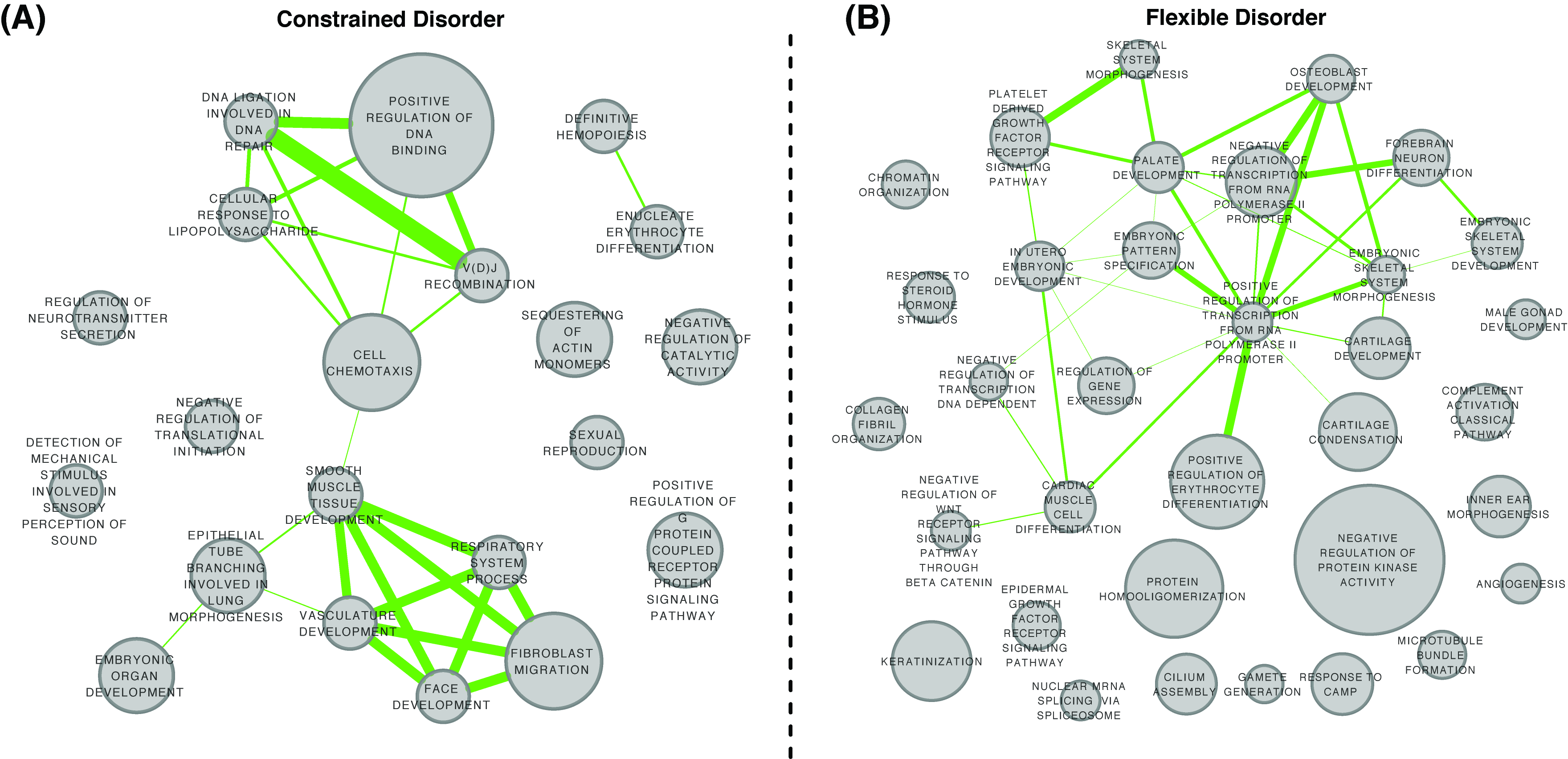

Supplement: Figure S1 — Each network is a representation of the GO terms over-represented in the sets of proteins enriched in (A) Constrained disorder, (B) Flexible disorder. Each node represents a GO terms, its size indicating the significance of the enrichment (the bigger the node, the more significant the enrichment). Edges represent overlap between two GO terms (Overlap coefficient). (TIF) [file pcbi.1003030.s001.tif]

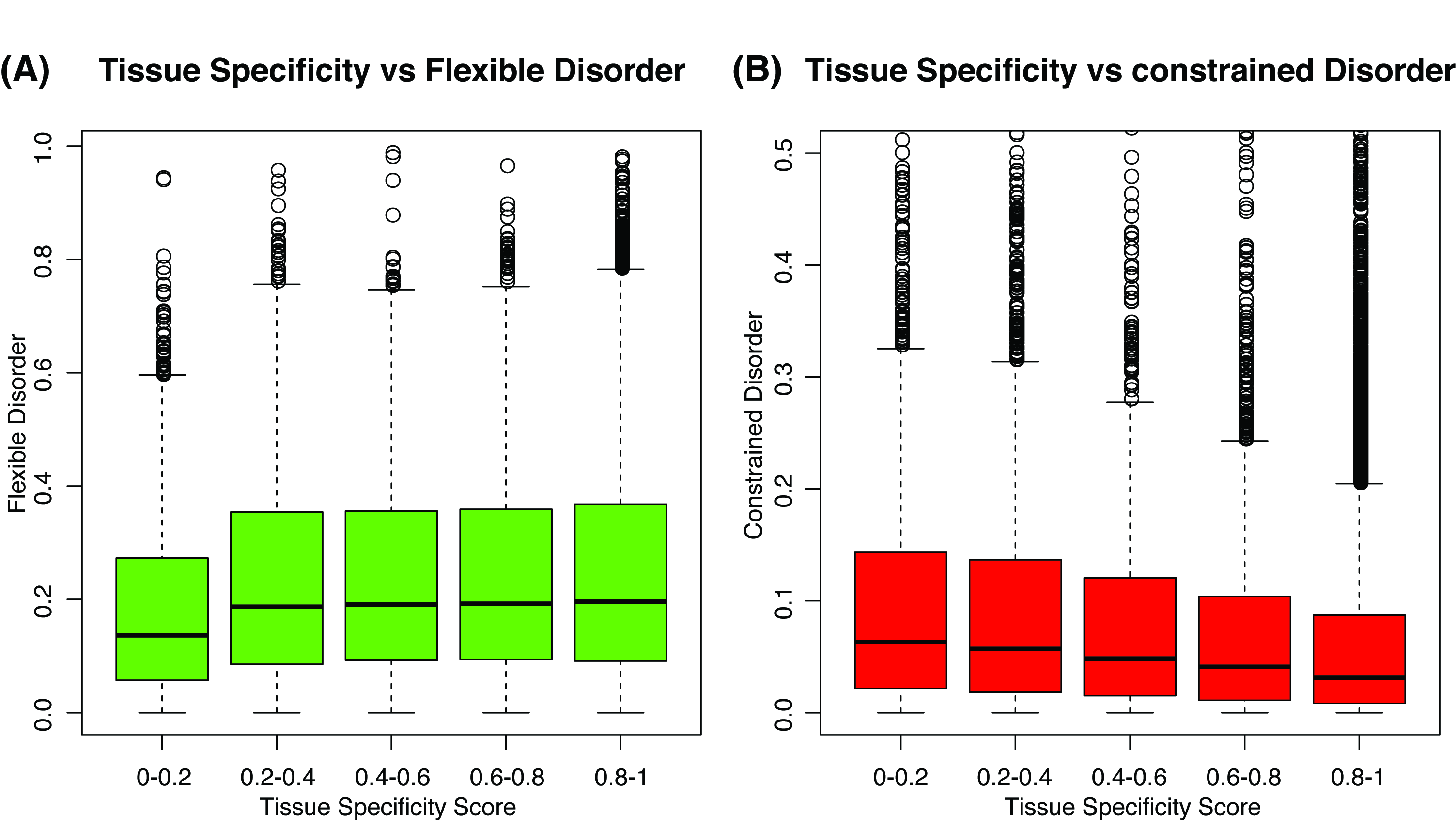

Supplement: Figure S2 — The boxplots show the correlation between the tissue specificity of the gene and the portion of (A) flexible disorder and (B) constrained disorder. All genes are binned into 5 different bins depending on the tissue specificity score. (TIF) [file pcbi.1003030.s002.tif]

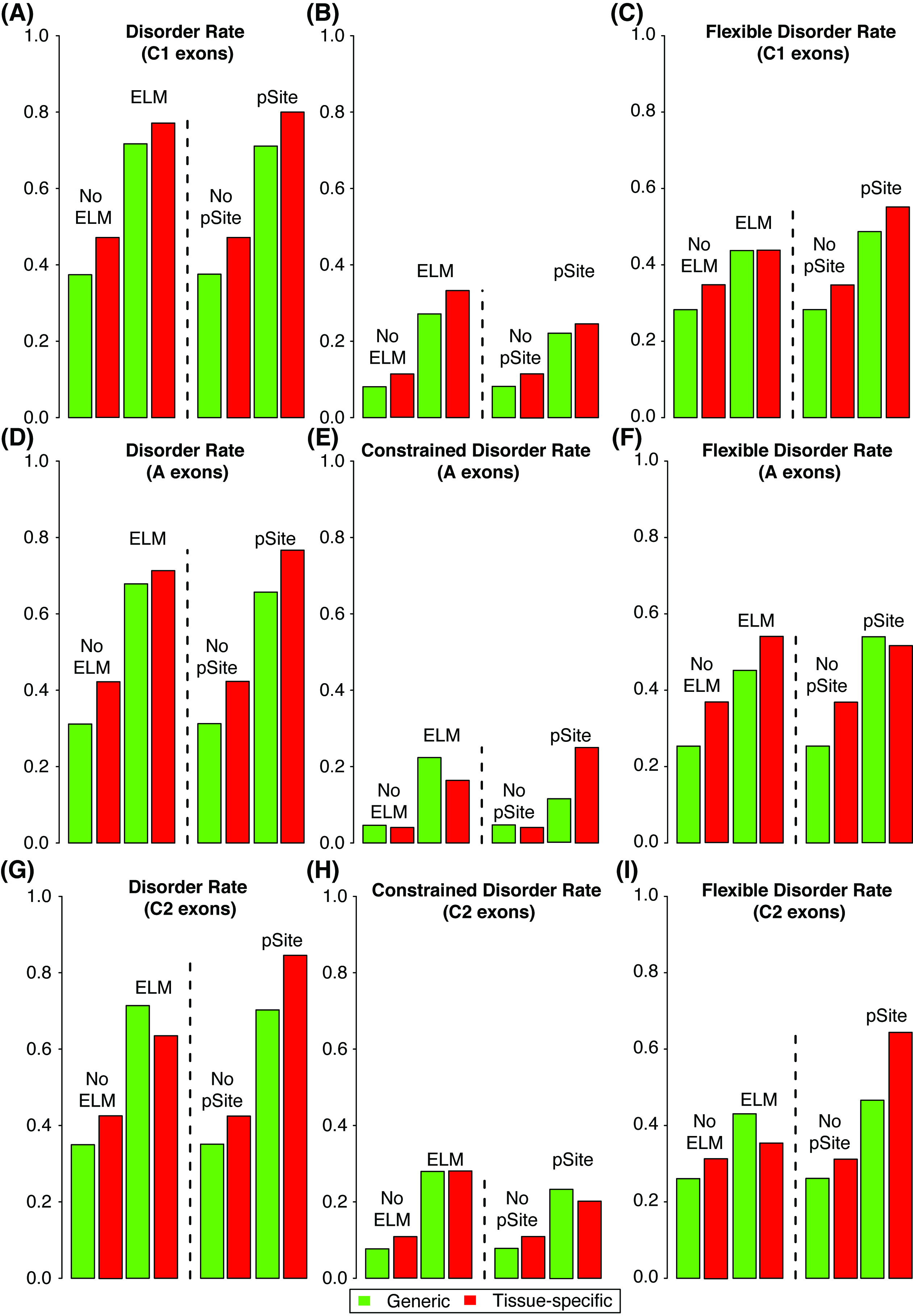

Supplement: Figure S3 — The enrichment of disorder, constrained disorder, and flexible disorder in different types of exons is largely driven by phosphosites and ELMs. (A–C) C1 exons, (D–F) A exons, (G–I) C2 exons. (TIF) [file pcbi.1003030.s003.tif]

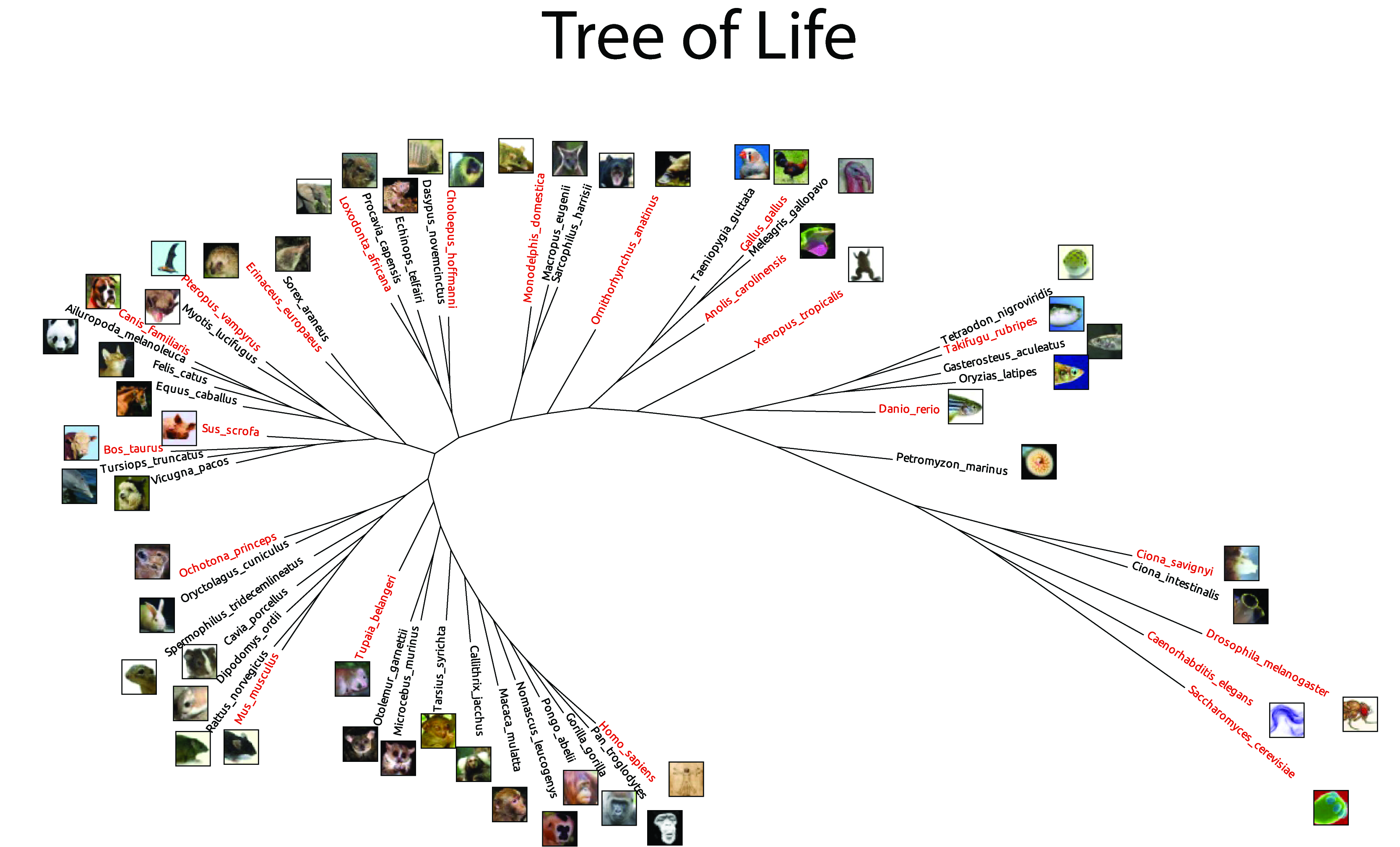

Supplement: Figure S4 — The species chosen for analyses are labeled red in the phylogenetic tree. (TIF) [file pcbi.1003030.s004.tif]

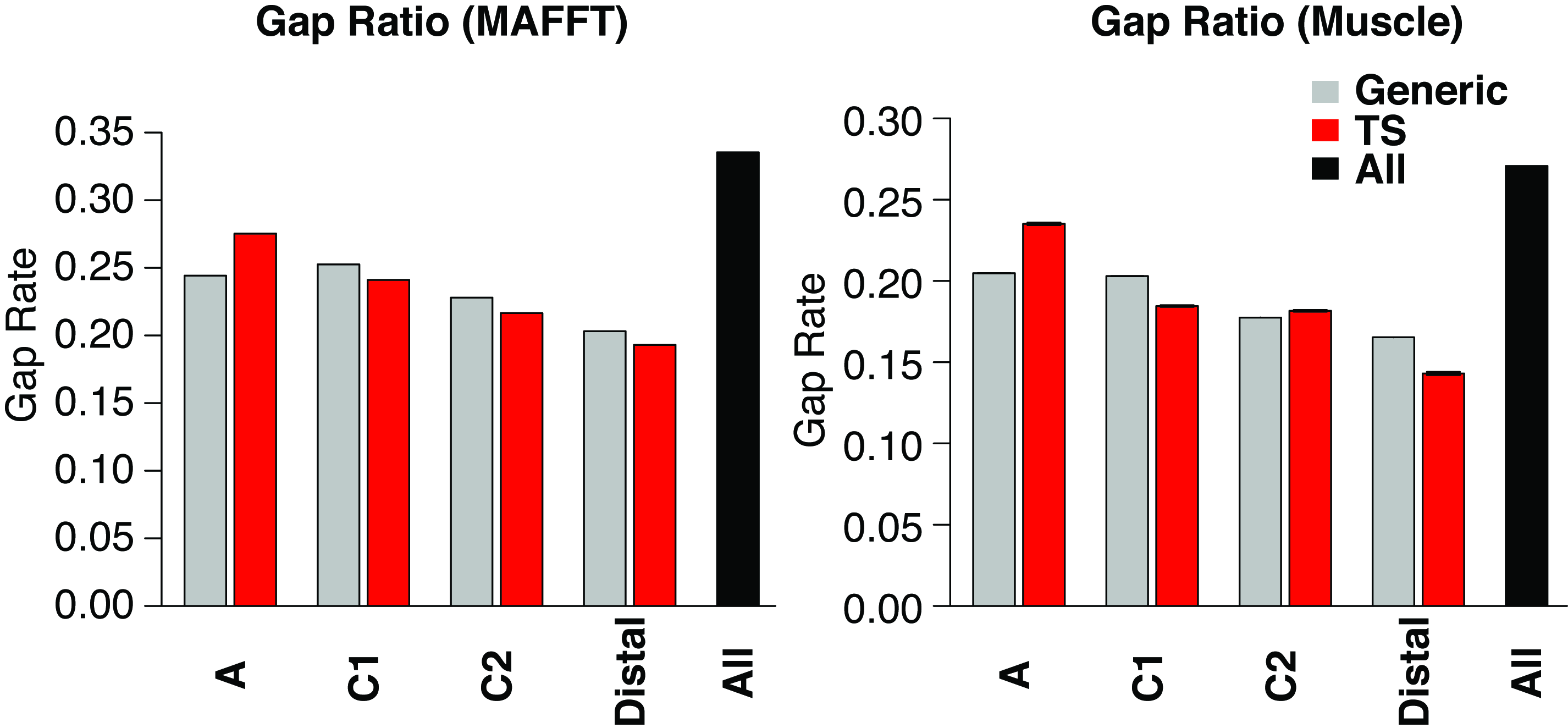

Supplement: Figure S5 — Ratio of gaps for each region types based on the orthologs alignments generated by (A) MAFFT and the (B) the MUSCLE multiple sequence aligners. Gap rate is calculated as average gap ratio within the exon/region, which is calculated as the number of gaps for a given site divided by number of species in the alignment. (TIF) [file pcbi.1003030.s005.tif]

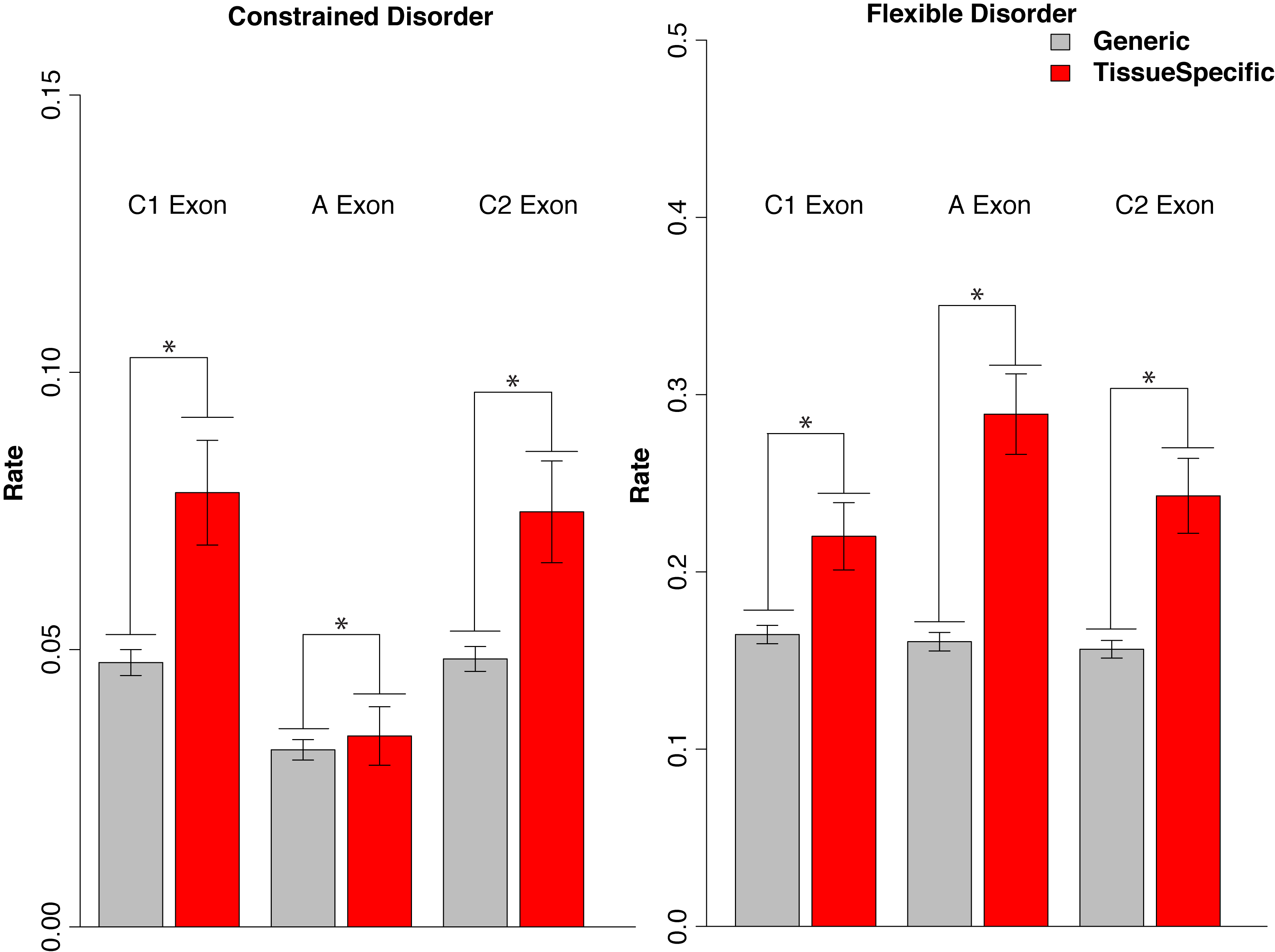

Supplement: Figure S6 — Conserved disorder rate analysis using the MUSCLE and IUPred tool combination. (A) Constrained disorder is only enriched in flanking (C1 and C2) exons (P<3.62e-08 for C1 and P<0.0003 for C2). The tissue-specific alternatively spliced exons are not enriched in constrained disorder. (B) Flexible disorder is highly enriched in tissue-specific A exons (P<6.91e-08). (TIF) [file pcbi.1003030.s006.tif]
