# Supplementary material for: Distinct Types of Disorder in the Human Proteome: Functional Implications for Alternative Splicing
Source: PLoS Comput Biol. 2013 Apr 25;9(4):e1003030. doi: 10.1371/journal.pcbi.1003030 (PMC3635989; doi:10.1371/journal.pcbi.1003030)
Supplement: Text S1 — Alternative alignments and disorder prediction methodology. Results obtained from re-implementing our pipeline with MUSCLE [42] and IUPred [54] tool combination. (DOCX) [file pcbi.1003030.s010.docx]

**Distinct types of disorder in the human proteome: functional implications for alternative splicing**

**Supplement**

Recep Colak^1,2,4*^, TaeHyung Kim^1,2,4*^, Magali Michaut^1,2^, Mark Sun^1,2,4^, Manuel Irimia^1,2^, Jeremy Bellay^5^, Chad L. Myers^5^, Benjamin J Blencowe^1,2^¶ and

Philip M. Kim^1,2,3,4^¶

^1^The Donnelly Centre

^2^Banting and Best Department of Medical Research

^3^Department of Molecular Genetics

^4^Department of Computer Science

University of Toronto

Toronto, ON M5S 3E1

Canada

^5^Department of Computer Science and Engineering

University of Minnesota

Minneapolis, MN

USA

^*^These authors contributed equally to this work.

**¶** To whom correspondence should be addressed:

Tel: +1 416 946 3419; Fax: +1 416 978 8287;

Email: [pi@kimlab.org](mailto:pi@kimlab.org)

Running title: Conserved disorder in higher eukaryotes

Character count: ~ 35,000

**Supplement**

**Text S1. Alternative alignment and disorder prediction methodology**

To account for potential biases introduced by the alignment algorithm and the disorder prediction algorithms, i.e. dependency on PSI-blast, we did all our analysis on two distinct and independent pipelines. In addition to the MAFTT aligner [1] and DisoPred2 [2]disorder prediction tool combination, we performed all our analysis with MUSCLE aligner [3] and IUPred disorder prediction tool [4]combination as well. For this, we first aligned all orthologs from the selected species with MUSCLE multiple sequence aligner. Even though MUSCLE seems to be more conservative with gaps compared to MAFFT aligner, overall both tools resulted highly similar alignments. Moreover, to discover potential biases caused by non-conserved alternative exons (A exons) that would confound the flexible vs constrained disorder ratio analysis; we checked the gap ratios in every exon type and whole proteins. Both aligners gave virtually the same result, with MUSCLE giving reduced rates of gaps (See Figure 5S). As such we concluded that the specific choice of aligner does not affect our subsequent analysis. We also observed that A exons are less conserved (contain more gaps in the alignment) compared to both C1, C2 and distal exons, which are exons from alternatively spliced transcripts that are not far away from the splicing site (See Figure 5S). More importantly, we observed that tissue specific exons does not necessarily contain more gaps than general exon types except the A exon. However even for A exon, the difference is minor (<%3), and is far below the difference we observe in flexible and constrained disorder rates. For example, tissue specific A exons are >15% more flexibly disordered compared to general A exons. As such, we also conclude that distinct evolutionary forces acting on A, C1 and C2 exons do not confound our analysis. Next, we performed disorder prediction using IUPred, which does not depend on PSI-blast and hence is not affected by potential biases that naturally exist in the structural databases. It turned out that all top performers in CASP9 disorder prediction competition [5] use either PSI-blast or depend on it due to hybrid/ensemble prediction approaches. Even though IUPred is not among the top performers, it is one of best predictors known to have low false positive rate. Moreover, IUPred is fast and can be run on proteome of all organisms in a short amount time enabling us to perform end-to-end analysis. Finally, Figure 6S reveals that MUSCLE and IUPred tool combination give qualitatively the same results as MAFTT and DisoPred2 tool combination shown in Figure 3.

Last but not least, we also analyzed the bias in flexible vs. constrained disorder calls that might be caused by increased rates of gapped alignments as a by-product of difference in exon sets of the aligned orthologs. For this, we systematically removed peptide segments from all conserved disorder related analysis at various gap thresholds. We start with introducing two parameters, *the gap calling threshold*, which is the threshold after which the percentage of gaps at a given position results in calling the given position a **gap** and, *gapped window calling threshold*, which is the threshold after which the average gap rate at a position’s 10aa neighborhood results in the area to be called a gapped window and removed from subsequent analysis. To illustrate, if gap-calling threshold is 0.3 and a given position has 6 gaps out of the 19 orthologs, then we call this position as gap and next check its neighborhood. If the same trend holds within 10aa neighborhood, then we remove this peptide segment from all subsequent analysis. Our reasoning for looking at neighborhood before removing a position is that we want to differentiate gaps caused by small indels and mutations from gaps caused by exon content difference, which would naturally come in a larger number of gapped positions, i.e. gap windows. With this scheme, we analyzed the entire spectrum of the two parameters (See Figure 7S) and discovered that the flexible disorder of tissue specific A exons changes in complete agreement with that of the general A exons, while always maintaining a significant difference. For constrained disorder rate, however, we see that both sets are close to each other over the entire spectrum, in complete agreement with our earlier analysis (See Figure 3). In summary, these experiments prove that gaps are important and quantitative results change according to gap content, though TS and general A exons do not show any significant difference in terms of exon contents to their respective orthologs. Our results are also consistent across the two alignments and disorder calling tool combinations.

References

1. Katoh K, Toh H (2010) Parallelization of the MAFFT multiple sequence alignment program. Bioinformatics 26: 1899–1900.

2. Ward JJ, McGuffin LJ, Bryson K, Buxton BF, Jones DT (2004) The DISOPRED server for the prediction of protein disorder. Bioinformatics 20: 2138–2139. doi:10.1093/bioinformatics/bth195.

3. Edgar RC (2004) MUSCLE: multiple sequence alignment with high accuracy and high throughput. Nucleic acids research 32: 1792–1797. doi:10.1093/nar/gkh340.

4. Dosztányi Z, Csizmok V, Tompa P, Simon I (2005) IUPred: web server for the prediction of intrinsically unstructured regions of proteins based on estimated energy content. Bioinformatics.

5. Monastyrskyy B, Fidelis K, Moult J, Tramontano A, Kryshtafovych A (2011) Evaluation of disorder predictions in CASP9. Proteins 79: 107–118. doi:10.1002/prot.23161.
