# Supplementary material for: Distinct Types of Disorder in the Human Proteome: Functional Implications for Alternative Splicing
Source: PLoS Comput Biol. 2013 Apr 25;9(4):e1003030. doi: 10.1371/journal.pcbi.1003030 (PMC3635989; doi:10.1371/journal.pcbi.1003030)
Supplement: Text S2 — A note on results of Buljan et al [9]. Comparison of our ELM enrichment against the results reported in Buljan et al [9]. (DOCX) [file pcbi.1003030.s011.docx]

**Distinct types of disorder in the human proteome: functional implications for alternative splicing**

**Supplement**

Recep Colak^1,2,4*^, TaeHyung Kim^1,2,4*^, Magali Michaut^1,2^, Mark Sun^1,2,4^, Manuel Irimia^1,2^, Jeremy Bellay^5^, Chad L. Myers^5^, Benjamin J Blencowe^1,2^¶ and

Philip M. Kim^1,2,3,4^¶

^1^The Donnelly Centre

^2^Banting and Best Department of Medical Research

^3^Department of Molecular Genetics

^4^Department of Computer Science

University of Toronto

Toronto, ON M5S 3E1

Canada

^5^Department of Computer Science and Engineering

University of Minnesota

Minneapolis, MN

USA

^*^These authors contributed equally to this work.

**¶** To whom correspondence should be addressed:

Tel: +1 416 946 3419; Fax: +1 416 978 8287;

Email: [pi@kimlab.org](mailto:pi@kimlab.org)

Running title: Conserved disorder in higher eukaryotes

Character count: ~ 35,000

**Supplement**

**Text S2. A note on results of Buljan et al.** [6]

Recently Buljan et al. reported that tissue specific alternative exons are enriched more with binding motifs compared to both general and constitutive A exons [6]. However, we would like to point out that what they measured is the percentage of A exons that have at least one binding site, which is different than ratio of amino acids in distinct exon types that fall into a binding motif (See Fig 4B). When we performed the same analysis as Buljan et al., namely measured the percentage of A exons with at least one binding site, we realized complete agreement with their finding using both their binding motif dataset and our internal ELM dataset (P< 0.001586 and P<0.12 respectively). Note however that, we deliberately chose to report our findings in terms of binding motif density as we believe it better reflects the real amount of regulatory activity.

References

6. Buljan M, Chalancon G, Eustermann S, Wagner GP, Fuxreiter M, et al. (2012) Tissue-Specific Splicing of Disordered Segments that Embed Binding Motifs Rewires Protein Interaction Networks. Molecular Cell 46: 871–883. doi:10.1016/j.molcel.2012.05.039.
